# Association of Timing of Adverse Childhood Experiences and Caregiver Support With Regionally Specific Brain Development in Adolescents

**DOI:** 10.1001/jamanetworkopen.2019.11426

**Published:** 2019-09-18

**Authors:** Joan L. Luby, Rebecca Tillman, Deanna M. Barch

**Affiliations:** 1 Department of Psychiatry (Child), School of Medicine, Washington University in St Louis, St Louis, Missouri; 2 Department of Psychiatry, School of Medicine, Washington University in St Louis, St Louis, Missouri; 3 Department of Psychological and Brain Sciences, Washington University in St Louis, St Louis, Missouri

## Abstract

**Importance:**

Few data are available to inform the associations and timing of the associations between adversity, caregiver support, and brain outcomes. Consideration of timing has important public health implications to inform more precise prevention strategies.

**Objective:**

To evaluate the timing and regional specificity of the association between adverse childhood experiences (ACEs) and caregiver support to structural development of limbic and striatal brain regions in middle childhood and adolescence.

**Design, Setting, and Participants:**

This 15-year developmental, neuroimaging cohort study included 211 children and their caregivers screened from day care centers and preschools in the St Louis, Missouri, metropolitan area during the preschool period, with an additional 4 waves of neuroimaging at school age through adolescence from November 14, 2007, to August 29, 2017. The cohort was oversampled for preschoolers with elevated symptoms of depression using a brief screener. Data analysis was performed from March 19, 2019, to July 26, 2019.

**Main Outcomes and Measures:**

Volumes in adolescence and developmental trajectories of volumes of the amygdala, hippocampus, caudate, subgenual cingulate, and insula during 4 waves of scanning; ACEs and observed caregiver support at preschool and school age; and volumes of amygdala, hippocampus, insula, and subgenual cingulate during 4 waves of scanning.

**Results:**

A total of 211 children (107 [50.7%] male) completed at least 1 scan. At preschool (mean [SD] age, 5.5 [0.8] years), ACE data were available for 164 children (84 [51.2%] male) and maternal support data for 155 children; at school age (mean [SD], 8.3 [1.2] years), ACE data were available for 172 children and maternal support data for 146 children. Unique patterns of the association between ACEs and support were found, with an association between the interaction of preschool ACEs and school-age support and the development of the hippocampus (*t* = −2.27; *P* = .02) and amygdala (*t* = −2.12; *P* = .04). A buffering hypothesis was not confirmed because high caregiver support was more strongly associated with the development of these regions only in the context of low ACEs. In contrast, preschool ACEs (*t* = −2.30; *P* = .02) and support (*t* = 2.59; *P* = .01) had independent associations with the development of the caudate.

**Conclusions and Relevance:**

The findings suggest that there are unique regional associations of support and adversity with key brain structures important for emotional regulation. Results may inform the timing and potential targets of preventive action for the range of poor developmental outcomes.

## Key Points

**Question:**

Is there developmental timing and regional specificity to the associations among adverse childhood experiences, caregiver support, and structural brain development in childhood?

**Findings:**

This cohort study of 211 children and their caregivers during 4 waves of neuroimaging and behavioral assessments from preschool to adolescence found an association between the interaction of preschool adverse childhood experiences and support during school age and the structural development of the amygdala and hippocampus, with volumes of these regions being the largest in children with low adverse childhood experiences and high caregiver support. In contrast, both preschool adverse childhood experiences and support were independently associated with the development of the caudate.

**Meaning:**

Findings may inform the timing and targets for prevention of poor outcomes related to exposure to adversity.

## Teaser

This cohort study assesses the timing and regional specificity of the association of adverse childhood experiences and caregiver support with structural development of limbic and striatal brain regions in middle childhood and adolescence.

## Introduction

Increasing evidence of the associations between early-life experience and human brain development has emerged during the past 2 decades.^1^ Building on animal studies and retrospective data in humans, several prospective neuroimaging studies^2,3,4,5,6^ have further elucidated the negative associations of varying forms of adversity and the positive associations of nurturing caregiving in early childhood with the key brain regions critical for adaptive functioning. Related to these findings, sensitive periods during which environmental factors have a more powerful association with brain development have been found and are known to vary by region.^7^ These risk and protective factors and their associations with the function and structure of limbic and frontal regions have been directly associated with mental health outcomes.^8,9,10^ These studies underscore the association of potentially modifiable environmental factors with childhood brain development and represent a paradigm change in the understanding of the development of brain regions subserving key emotional and cognitive capacities previously believed to be largely genetically driven.

Prospective studies^10,11,12,13,14^ have provided evidence that early-childhood adversity, often manifest as exposure to poverty and/or experiences of abuse and neglect, are associated with the structural development of limbic regions, including the hippocampus and amygdala. Although less well replicated, data also suggest similar associations in the basal ganglia,^12,15,16^ with retrospective and some prospective data also suggesting associations with the dorsal prefrontal cortex, anterior cingulate, and subgenual cingulate.^9,14,15,17,18,19,20^

Although these negative associations between adversity and neural development are increasingly clear, there is also evidence that supportive caregiving has important positive associations with brain development and may in some situations serve as a protective or buffering factor that mitigates this risk. Data from the longitudinal developmental neuroimaging study^21,22^ examined here have demonstrated positive associations between early caregiver support and later childhood hippocampal volume, with preschool identified as a sensitive period. In the same study sample, negative associations between poverty and limbic outcomes were mediated by caregiving support and stressful life events.^23^ Whittle and colleagues^9^ reported positive associations between supportive caregiving and dorsal frontal lobe and amygdala development in adolescents exposed to poverty. Positive associations between caregiver support and resting state connectivity have also been reported in a longitudinal study^24^ of children raised in poverty. However, more information is needed on the interactive effects of adversity and caregiver support.

The extant literature offers few data to inform the associations and timing of the associations between adversity and caregiver support and brain outcomes. Consideration of timing has important public health implications to inform more precise prevention strategies. Myelination and experience-dependent processes, such as pruning, vary by brain region, with different areas reaching peak synaptogenesis at different times, suggesting that there may be developmental specificity to associations of adversity and nurturance that varies by region.^25^ Prospective data that inform the developmental timing of when adversity and support have their most powerful associations with the development of key brain regions and if and how they might interact are needed. We tested the following hypotheses: (1) adversity and caregiver support will each have independent associations with brain development; (2) the timing (preschool, school age) of adversity and caregiver support will influence associations with brain development, with stronger associations in the preschool period; and (3) caregiver support will moderate (interact with) the association between early adversity and brain development, such that among children with higher caregiver support there will be less of a negative association between early adversity and brain development in cortical and subcortical regions known to be related to early adversity.

## Methods

### Participants

Data from the Preschool Depression Study, a 15-year (June 1, 2003, onward), neuroimaging cohort study that began when participants were preschoolers, were used for this analysis (eFigure in the Supplement gives a study overview). This study included 211 children and their caregivers screened from day care centers and preschools in the St Louis, Missouri, metropolitan area during the preschool period, with an additional 4 waves of neuroimaging at school age through adolescence from November 14, 2007, to August 29, 2017. During 1 preschool and 2 school-age waves, observational measures of parenting during mildly stressful laboratory tasks were obtained. During each study wave (ages during waves are given in the eFigure in the Supplement), measures of experiences of adversity since the last wave were collected. Neuroimaging was conducted with child participants during 4 waves that spanned middle childhood to young adulthood completed to date. Data analysis was performed from March 19, 2019, to July 26, 2019. Written parental consent and child assent or consent (depending on age) to a protocol approved by the institutional review board at Washington University in St Louis were obtained before study participation. All data were deidentified. This report followed the Strengthening the Reporting of Observational Studies in Epidemiology (STROBE) reporting guideline.

### Measures

#### Adverse Childhood Experiences 

We created adverse childhood experience (ACE) scores using variables from the life events section of the Preschool Age Psychiatric Assessment (PAPA)^26^ or Child and Adolescent Psychiatric Assessment (CAPA),^27^ parental psychopathologic variables from the Family Interview for Genetic Studies,^28^ and exposure to poverty based on family income to needs ratio at each assessment wave. The ACE scores were similar to those previously reported.^29^ The PAPA was conducted at each assessment that occurred between the ages of 3 and 7 years based on parent report, and the CAPA was used at 8 years of age based on parent report; both parent and child reports were obtained for 9 years or more. The ACE variable was adapted from the original Felitti definition but also included exposure to poverty because of its established role in brain development. Poverty was defined as an income to needs ratio less than 1, in accordance with federal guidelines. The variables included in the ACE sum scores and methods used to create the sum scores are given in eTable 1 in the Supplement.

#### Preschool and School-age Maternal Support

Maternal support in the present study was defined as the degree to which caregivers (92% mothers) approached and interacted with their children with positive regard and nurturance as well as their efforts to be emotionally and developmentally supportive. Preschool maternal support was measured during the second annual study wave when children were aged 3 to 6 years; their caregivers engaged in a mildly stressful laboratory task. This task, known as the waiting task, which was designed to evoke mild dyadic stress, required the child to wait for 8 minutes before opening a brightly wrapped gift within reach while the parent completed questionnaires. Staff trained on reliability coded the interaction for supportive caregiving strategies used to help regulate the child’s frustration in the face of thwarted ability to open the gift. Prior findings^21,22,30,31,32^ suggest that caregiving support coded during this task has good psychometric properties.

A different developmentally appropriate mildly stressful task, the puzzle task, was completed by children and their caregivers during the third annual study wave (when children were 6-7 years of age) and again during their fifth assessment wave 4 to 5 years later (8-12 years of age) as a measure of school-age maternal support. The child was asked to attempt to put together a puzzle without being able to see the pieces while relying on the parent (who could see the puzzle board and placement of the child’s hands) for instructions. Parents gave the child instructions on where to place each piece, and the dyad earned a prize if they finished the puzzle in 5 minutes. Both of these measures of maternal support have been previously published.^21^

#### Magnetic Resonance Imaging Acquisition

Structural images were collected as part of longer scan sessions that also included acquisition of task-based and functional connectivity data. For the first 3 waves, imaging data were collected using a 3-T Siemens TIM TRIO. T1-weighted structural images were acquired in the sagittal plane using 2 magnetization-prepared rapid gradient-echo (MPRAGE) 3-dimensional sequences (repetition time, 2400 milliseconds; echo time, 3.16 milliseconds; flip angle, 8°; 176 sections; field of view, 256 mm; 1.0-mm^3^ voxels). For scan 4, we used a 3-T Siemens PRISMA with a 32-channel head coil using Human Connectome Project–style acquisitions,^33^ using a sagittally acquired MPRAGE 3-dimensional sequence (repetition time, 2300 milliseconds; echo time, 3.16 milliseconds; flip angle, 8°; 160 sections; field of view, 256 mm; 1.0-mm^3^ voxels). Correlations of whole brain volumes across scans were high (0.954 to 0.981 for total gray-matter volume, 0.984 to 0.991 for supratentorial volume, and 0.917 to 0.999 for intracranial volume), suggesting that scanner changes should not influence our ability to examine associations of ACEs and caregiver support to brain development.

Brain volumes were generated using the FreeSurfer software, version 5.3 processing stream described by Luby et al^29^ (further details are given in eTable 1 in the Supplement). Data on the volume of the left and right hippocampus, amygdala, caudate, nucleus accumbens, and putamen in the participant’s native space were obtained using the FreeSurfer aseg.stats report.^33,34^ Volumes of the anterior insula, subgenual cingulate, dorsolateral prefrontal cortex, dorsal anterior cingulate, and rostral cingulate were taken from Destrieux Atlas (eTable 1 in the Supplement).^35^ We did not have a priori hypotheses about left or right hemispheres, and thus, we averaged the 2. These regions of interest were chosen based on the evidence in the extant literature reviewed in the Introduction section. Whole brain volume was used as a covariate to assess specificity.

### Statistical Analysis

We used a hierarchical analytical approach to identify brain regions with associations with ACEs and caregiver support. We started with 4 sets of general linear models (GLMs) evaluating the volumes of the regions of interest at scan 4, when participants were adolescents or young adults. Each set included covariates of scan 4 (age, sex, and whole brain volume) and 1 of the 4 following variables of interest: ACEs and maternal support at preschool and school age. The results were false discovery rate (FDR) corrected within each set of GLMs, with an FDR with a 2-sided *P* < .05 indicating significance. In cases in which an independent variable was associated with brain volume at one age but not another, an additional GLM was conducted that included both age periods to assess the specificity of the association with the one age period.

Any variables significant after FDR correction in the GLMs described above were then included in multilevel models (MLMs) to examine whether ACEs and/or maternal support at preschool and/or school age were significantly associated with the trajectory of brain volumes across scans 1 to 4. The MLMs used random intercept and slope components and unstructured covariance. Time was defined as age at scan, which was centered at 12 years of age, and the covariates were sex and whole brain volume. When the interaction between an independent variable and age was not significant (*P* ≥ .05), the variable was removed from the model to test for main effects. To test hypotheses about interactions between ACEs and maternal support, 3-way interactions among ACEs, maternal support, and age were tested and included in the final models when significant.

## Results

A total of 211 children completed at least 1 scan. At preschool (mean [SD] age, 5.5 [0.8] years), data on ACEs were available for 164 children (84 [51.2%] male) and maternal support data for 155 children; at school age (mean [SD], 8.3 [1.2] years), data on ACEs were available for 172 children and maternal support data for 146 children. Correlations among these 4 variables are given in eTable 2 in the Supplement. Table 1 provides demographic and diagnostic characteristics of the samples included in each of the MLMs. Participants were not included in analyses if they had unusable scan data at all scan waves or were missing data on preschool ACEs because this was an independent variable in every MLM. Missing data were not imputed. Comparisons between participants included (n = 164) and not included (n = 47) in analyses are presented in eTable 3 in the Supplement.

**Table 1.** Characteristics of Participants Included in MLM Analyses

Characteristic	MLM				
	Insula Volume (n = 138)^a^	Hippocampus Volume (n = 133)^b^	Amygdala Volume (n = 141)^c^	Subgenual Cingulate Volume (n = 164)^d^	Caudate Volume (n = 151)^e^
Male sex, No. (%)	75 (54.4)	69 (51.9)	76 (53.9)	84 (51.2)	77 (51.0)
Race/ethnicity, No. (%)					
White	71 (51.5)	70 (52.6)	72 (51.1)	88 (53.7)	83 (55.0)
African American	52 (37.7)	48 (36.1)	54 (38.3)	57 (34.8)	50 (33.1)
Other	15 (10.9)	15 (11.3)	15 (10.6)	19 (11.6)	18 (11.9)
Scan age, mean (SD), y					
Wave 1	10.53 (1.06)	10.53 (1.06)	10.53 (1.05)	10.28 (1.24)	10.29 (1.25)
Wave 2	12.03 (1.02)	12.04 (1.01)	12.03 (1.01)	11.82 (1.16)	11.83 (1.15)
Wave 3	13.35 (1.07)	13.38 (1.07)	13.36 (1.06)	13.10 (1.20)	13.13 (1.22)
Wave 4	16.61 (0.95)	16.62 (0.95)	16.60 (0.96)	16.46 (1.00)	16.50 (0.99)
Scan 1 income to needs ratio	1.72 (0.99)	1.74 (0.99)	1.70 (0.99)	1.71 (1.00)	1.75 (1.00)
Maternal support, mean (SD)^f^					
Preschool	11.59 (8.25)	11.53 (8.24)	11.53 (8.24)	11.87 (8.57)	11.87 (8.57)
School-age	30.78 (9.06)	30.88 (9.00)	30.72 (9.09)	30.72 (9.09)	30.88 (9.00)
ACEs, median (IQR), No.					
Preschool	0.02 (−0.61 to 0.63)	−0.02 (−0.61 to 0.63)	0.02 (−0.61 to 0.63)	0.01 (−0.61 to 0.63)	−0.02 (−0.61 to 0.65)
School-age	−0.09 (−0.51 to 0.78)	−0.09 (−0.51 to 0.78)	−0.09 (−0.51 to 0.78)	−0.21 (−0.51 to 0.74)	−0.19 (−0.51 to 0.74)

Abbreviations: ACEs, adverse childhood experiences; IQR, interquartile range; MLM, multilevel model.

^a^ Preschool ACEs, school-age ACEs, and school-age maternal support data available.

^b^ Preschool ACEs, preschool maternal support, and school-age maternal support data available.

^c^ Preschool ACEs and school-age maternal support data available.

^d^ Preschool ACEs data available.

^e^ Preschool ACEs and preschool maternal support data available.

^f^ Information on measurement of maternal support is given in the Measures subsection of the Methods section.

### Preschool or School-age ACEs and Adolescent and Young Adult Brain Volume

As shown in eTable 4 in the Supplement, after FDR, higher preschool ACE scores were significantly associated with decreased insula, hippocampus, amygdala, subgenual cingulate, and caudate volume at scan 4. Greater school-age ACEs were significantly associated only with decreased insula volume. As detailed in eTable 5 in the Supplement, when both preschool and school-age ACEs were included in these models, preschool ACEs continued to have the only significant association with the brain volume for amygdala (mean [SE] estimate, −0.067 [0.024]; *t* = −2.77; *P* = .007), subgenual cingulate (mean [SE] estimate, −0.121 [0.060]; *t* = −2.03; *P* = .04), and caudate (mean [SE] estimate, −0.165 [0.065]; *t* = −2.55; *P* = .01).

### Preschool or School-age Support and Adolescent and Young Adult Brain Volume

After FDR correction, greater preschool maternal support was significantly associated with increased hippocampus (mean [SE] estimate, 0.017 [0.005]; *t* = 3.38; FDR *P* = .005) and caudate (mean [SE] estimate, 0.019 [0.005]; *t* = 3.57; FDR *P* = .005) volume at scan 4, and greater school-age maternal support was significantly associated with increased insula (mean [SE] estimate, 0.011 [0.004]; *t* = 2.72; FDR *P* = .04), hippocampus (mean [SE] estimate, 0.015 [0.005]; *t* = 3.03; FDR *P* = .03), and amygdala (mean [SE] estimate, 0.005 [0.002]; *t* = 2.58; FDR *P* = .04) volume at scan 4 (eTable 6 in the Supplement). As detailed in eTable 7 in the Supplement, when both preschool and school-age maternal support was included, maternal support during preschool continued to have the only significant association with scan 4 caudate volume (mean [SE] estimate, 0.019 [0.006]; *t* = 2.94; *P* = .004), whereas only school-age maternal support was significantly associated with scan 4 insula (mean [SE] estimate, 0.011 [0.005]; *t* = 2.36; *P* = .02) and amygdala (mean [SE] estimate 0.005 [0.002]; *t* = 1.97; *P* = .05) volume.

### ACEs and Maternal Support and Trajectories of Brain Volume

#### Insula Volume Trajectories

Greater preschool (mean [SE] estimate, −0.123 [0.033]; *t* = −3.78; FDR *P* = .002) and school-age (mean [SE] estimate, −0.106 [0.032]; *t* = −3.35; FDR *P* = .01) ACE scores and less school-age maternal support (mean [SE] estimate, 0.011 [0.004]; *t* = 2.72; FDR *P* = .04) were each significantly associated with reduced insula volume at scan 4. As indicated in Table 2, when all 3 of these independent variables were included in an MLM of insula volume across scans 1 to 4, only the main association of preschool ACEs was significant (mean [SE] estimate, −0.0626 [0.0296]; *t* = −2.11; *P* = .04), with higher preschool ACEs associated with smaller insula volume (smaller intercept). The interactions between variables and age (slope) were not significant and not included in the final model.

**Table 2.** Multilevel Models of Brain Volumes Across Scans 1 to 4 by ACEs and Maternal Support Covarying for Sex and Whole Brain Volume

Variable	Estimate (SE)	95% CI of Estimate	*t* Value	*P* Value
**Insula Volume (n = 138)**				
Intercept	3.3800 (0.0306)	3.3195 to 3.4405	110.54	<.001
Female	0.0356 (0.0473)	−0.0578 to 0.1289	0.75	.45
Whole brain volume	0.0024 (0.0002)	0.0020 to 0.0028	11.65	<.001
Age	−0.0329 (0.0033)	−0.0393 to −0.0264	−10.05	<.001
Preschool ACEs	−0.0626 (0.0296)	−0.1211 to −0.0041	−2.11	.04
School-age ACEs	−0.0059 (0.0281)	−0.0614 to 0.0497	−0.21	.84
School-age maternal support	0.0007 (0.0025)	−0.0043 to 0.0056	0.28	.78
**Hippocampus Volume (n = 133)**				
Intercept	4.0867 (0.0405)	4.0065 to 4.1669	100.81	<.001
Female	0.0474 (0.0600)	−0.0712 to 0.1660	0.79	.43
Whole brain volume	0.0024 (0.0003)	0.0018 to 0.0029	8.41	<.001
Age	0.0855 (0.0045)	0.0766 to 0.0945	18.93	<.001
Preschool ACEs	0.0005 (0.0290)	−0.0567 to 0.0577	0.02	.99
Preschool maternal support	0.0053 (0.0036)	−0.0019 to 0.0125	1.46	.15
School-age maternal support	0.0047 (0.0034)	−0.0019 to 0.0113	1.40	.16
Preschool ACEs × school-age maternal support	−0.0004 (0.0030)	−0.0062 to 0.0055	−0.13	.90
Preschool ACEs × age	−0.0133 (0.0045)	−0.0222 to −0.0043	−2.92	.004
School-age maternal support × age	−0.0002 (0.0005)	−0.0012 to 0.0008	−0.32	.75
Preschool ACEs × school-age support × age	−0.0011 (0.0005)	−0.0020 to −0.0001	−2.27	.02
**Amygdala Volume (n = 141)**				
Intercept	1.6162 (0.0171)	1.5824 to 1.6501	94.39	<.001
Female	−0.0251 (0.0257)	−0.0758 to 0.0256	−0.98	.33
Whole brain volume	0.0008 (0.0001)	0.0006 to 0.0011	7.30	<.001
Age	0.0068 (0.0026)	0.0017 to 0.0119	2.63	.01
Preschool ACEs	−0.0083 (0.0125)	−0.0330 to 0.0165	−0.66	.51
School-age maternal support	0.0017 (0.0014)	−0.0010 to 0.0044	1.28	.20
Preschool ACEs × school-age maternal support	0.0003 (0.0013)	−0.0022 to 0.0029	0.25	.80
Preschool ACEs × age	−0.0072 (0.0026)	−0.0124 to −0.0021	−2.78	.006
School-age maternal support × age	−0.0003 (0.0003)	−0.0009 to 0.0003	−1.07	.29
Preschool ACEs × school-age support × age	−0.0006 (0.0003)	−0.0011 to −0.0000	−2.12	.04
**Subgenual Cingulate Volume (n = 164)**				
Intercept	3.2617 (0.0389)	3.1849 to 3.3385	83.84	<.001
Female	0.0473 (0.0559)	−0.0629 to 0.1576	0.85	.40
Whole brain volume	0.0031 (0.0002)	0.0026 to 0.0035	12.43	<.001
Age	−0.0532 (0.0053)	−0.0637 to −0.0427	−9.98	<.001
Preschool ACEs	−0.0092 (0.0258)	−0.0601 to 0.0417	−0.36	.72
**Caudate Volume (n = 151)**				
Intercept	4.1313 (0.0518)	4.0289 to 4.2337	79.73	<.001
Female	0.0585 (0.0751)	−0.0897 to 0.2067	0.78	.44
Whole brain volume	0.0023 (0.0003)	0.0017 to 0.0029	7.41	<.001
Age	−0.0512 (0.0030)	−0.0571 to −0.0454	−17.25	<.001
Preschool				
ACEs	−0.0776 (0.0337)	−0.1441 to −0.0110	−2.30	.02
Maternal support	0.0105 (0.0040)	0.0025 to 0.0184	2.59	.01

Abbreviation: ACEs, adverse childhood experiences.

#### Hippocampus Volume Trajectories

Greater preschool ACEs (mean [SE] estimate, −0.102 [0.040]; *t* = −2.53; FDR *P* = .03) and less maternal support at preschool (mean [SE] estimate, 0.017 [0.005]; *t* = 3.38; FDR *P* = .005) and school age (mean [SE] estimate, 0.015 [0.005]; *t* = 3.03; FDR *P* = .03) were each significantly associated with reduced scan 4 hippocampal volume. As indicated in Table 2, when all these variables were in the model, the 3-way interaction among preschool ACEs, school-age maternal support, and age was significant (mean [SE] estimate, −0.0011 [0.0005]; *t* = −2.27; *P* = .02), indicating that preschool ACEs and school-age maternal support in interaction were associated with hippocampal trajectories over time.

**Figure 1.**
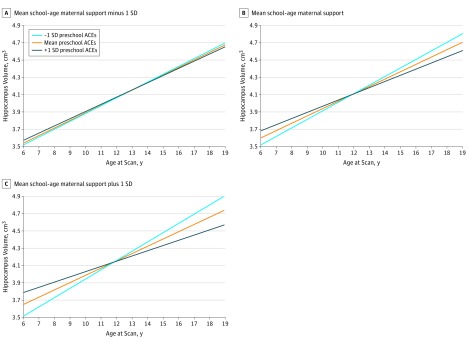
Estimated Trajectories of Hippocampus Volume by Preschool Adverse Childhood Experiences (ACEs) and School-age Maternal Support

To parse the source of this interaction and test our hypothesis about buffering, we centered the maternal support variable at −1 SD, mean, and +1 SD and examined the association between preschool ACEs and age. As shown in Figure 1, the interaction between preschool ACEs and age (eg, slope) was not significantly associated with hippocampus volume at low levels of maternal support (mean [SE] estimate, −0.0038 [0.0052]; *t* = −0.74; *P* = .46), but the association was significant at moderate (mean [SE] estimate, −0.0133 [0.0045]; *t* = −2.92; *P* = .004) and high (mean [SE] estimate, −0.0227 [0.0070]; *t* = −3.24; *P* = .002) levels of support. The largest estimated hippocampal volumes were at the combination of low values of preschool ACEs and high values of school-age maternal support.

#### Amygdala Volume Trajectories

**Figure 2.**
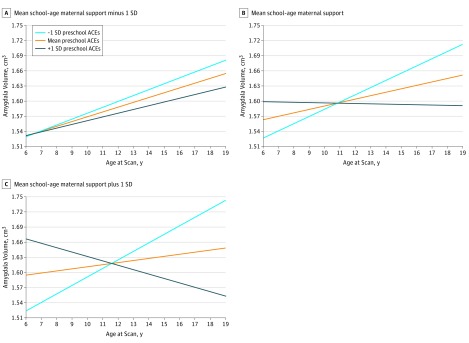
Estimated Trajectories of Amygdala Volume by Preschool Adverse Childhood Experiences (ACEs) and School-age Maternal Support

Preschool ACEs (mean [SE] estimate, −0.043 [0.017]; *t* = −2.58; FDR *P* = .003) and school-age maternal support (mean [SE] estimate, 0.005 [0.002]; *t* = 2.58; FDR *P* = .04) were each significantly associated with scan 4 amygdala volume. As indicated in Table 2, the 3-way interaction among preschool ACEs, school-age maternal support, and age was significant in the MLM (mean [SE] estimate, −0.0006 [0.0003]; *t* = −2.12; *P* = .04). To parse the source of this interaction and test our hypothesis about buffering, we again centered the maternal support variable at −1 SD, mean, and +1 SD and examined the association between preschool ACEs and age. As shown in Figure 2, the association between preschool ACEs and age (eg, slope) was not significantly associated with amygdala volume at low levels of maternal support (mean [SE] estimate, −0.0020 [0.0030]; *t* = −0.67; *P* = .50), but the association was significant at moderate (mean [SE] estimate, −0.0072 [0.0026]; *t* = −2.78; *P* = .006) and high (mean [SE] estimate, −0.0125 [0.0040]; *t* = −3.08; *P* = .002) levels of support. The largest estimated amygdala volumes were at the combination of low values of preschool ACEs and high values of school-age maternal support.

#### Subgenual Cingulate Volume Trajectories

Only preschool ACEs had a significant association with scan 4 subgenual cingulate volume (mean [SE] estimate, −0.106 [0.040]; *t* = −2.66; FDR *P* = .03). However, in the MLM across scans 1 to 4, preschool ACEs were not associated with the intercept or slope of subgenual cingulate over time.

#### Caudate Volume Trajectories

**Figure 3.**
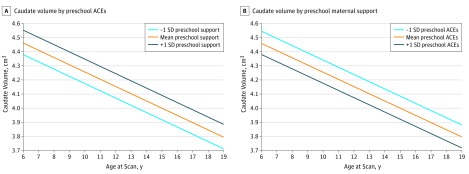
Estimated Trajectories of Caudate Volume by Preschool Adverse Childhood Experiences (ACEs) and Preschool Maternal Support

Both preschool ACEs (mean [SE] estimate, −0.101 [0.043]; *t* = −2.32; FDR *P* = .04) and maternal support (mean [SE] estimate, 0.019 [0.005]; *t* = 3.57; FDR *P* = .005) were significantly associated with scan 4 caudate volume. As indicated in Table 2, when both these independent variables were included in an MLM of caudate volume at scans 1 to 4, lower preschool ACEs (mean [SE] estimate, −0.0776 [0.0337]; *t* = −2.30; *P* = .02) and greater preschool maternal support (mean [SE] estimate, 0.0105 [0.0040]; *t* = 2.59; *P* = .01) were independently significantly associated with greater caudate volume (Table 2 and Figure 3). The interaction between these independent variables and age (slope) was not significant.

## Discussion

Findings from this longitudinal, developmental, neuroimaging cohort study replicate and extend findings in the extant literature regarding the associations of ACEs and maternal support with limbic and striatal brain regions previously reported to be associated with early psychosocial variables. Greater preschool ACEs were associated with lower volumes of hippocampus, amygdala, insula, subgenual cingulate, and caudate in late adolescence and early adulthood (scan 4), whereas school-age ACEs were only associated with the insula volume. With regard to maternal support, greater preschool support was associated with increased hippocampus and caudate volumes, whereas school-age support had positive associations with insula, hippocampus, and amygdala volumes. These findings provide new evidence, to our knowledge, of developmental specificity in the associations of psychosocial experience to brain development regionally when examined cross-sectionally.

Regionally varying patterns also emerged when trajectories of brain volumes during 4 scan waves were examined. The buffering hypothesis was not supported for any brain region. Instead, for hippocampus and amygdala, high support was associated with patterns of growth typically associated with better outcomes (eg, greater volumes) only in the context of low ACEs. This finding contrasts with previously reported buffering effects in the hippocampus^3^ but might be consistent with prior data suggesting that children realize their genetic potential at greater rates in the context of environmental advantage.^36^ A similar pattern emerged for amygdala volumes. These findings underscore the long-term deleterious associations between exposure to ACEs during the preschool period and the developmental trajectory of the hippocampus and amygdala but suggest that the positive association of maternal support with brain development is most apparent in the context of low preschool ACEs.

These findings differ somewhat from prior results^21^ in that both the hippocampus and amygdala volumes were negatively associated with ACEs experienced during the preschool period but positively associated with support at school age when all variables were in the model simultaneously, rather than preschool support, as in our prior hippocampal findings that did not consider ACEs. However, when considered independently, both preschool maternal support and school-age maternal support were associated with hippocampal volume at the last scan wave. This pattern suggests that preschool ACEs are associated with the variance in hippocampal volumes associated with preschool maternal support, whereas at school age, maternal support is associated with additional variance whether considered independently or in combination with other factors. Our findings with regard to amygdala volume also contrast with a previous report^37^ of institutionalized young children, which found associations with larger amygdala volumes. The type of neglect and deprivation experienced by institutionalized children may be qualitatively different from ACEs or the type of maternal support that we measured and therefore may have different associations with brain development. Taken together, these patterns suggest that adversity early in life may be associated with hippocampus and amygdala development but that caregiver support during childhood may be most positively associated with brain development in these regions in the context of low adversity. Findings therefore underscore the critical importance of determining whether preventing exposure to ACEs, particularly during the preschool period, may benefit childhood brain development.

In contrast, for the caudate, a key region known in reward processing and related risk for mood disorders, both lower preschool ACEs and greater preschool maternal support were independently associated with greater caudate volume, with no interaction between the 2. These findings suggest that for the caudate growth trajectory, preschool maternal support has positive associations with development even in the context of exposure to ACEs and that greater ACEs have negative associations with caudate development regardless of the level of maternal support.

For the insula, both preschool and school-age ACEs were associated with scan 4 volumes, but the only significant association with insula volume across all scans was that of preschool ACEs. Furthermore, despite the association of preschool ACEs with subgenual cingulate volume cross-sectionally at scan 4, there was no association with the trajectory of subgenual cingulate volume during the 4 scan waves. Thus, the associations of ACEs and support at different ages varied considerably across the range of brain regions previously found to be associated with these psychosocial factors.

Our findings suggest regional specificity to the timing of when ACEs and support have significant associations with limbic and striatal brain regions. Both low ACEs and high support were associated with healthy development of the hippocampus and amygdala, whereas each was independently associated with positive development of the caudate. The timing of the positive associations with support also varied by region, with school-age support having more of an association with hippocampus and amygdala volumes in the context of low ACEs, whereas preschool support and low ACEs were independently associated with caudate developmental trajectories. Furthermore, a different pattern was evident for the insula and subgenual cingulate, with preschool ACEs associated with the former and neither environmental variable associated with the trajectory of the latter.

### Limitations

This study has limitations. The study is limited by the fact that children were ascertained at preschool age and underwent scanning at school age. We therefore were not able to consider brain developmental outcomes earlier in life, when even stronger associations may be present. In addition, several other aspects of psychosocial experience were not measured, such as the home environment, community factors, and the presence of other caregivers, which could meaningfully be associated with brain development. In addition, our support variable focused primarily on maternal support, and more work is needed to understand the associations with paternal support.

## Conclusions

Our findings suggest that there is a nuanced and regionally specific pattern of the psychosocial factors associated with healthy development of limbic and striatal brain regions key to adaptive emotion processing. Future studies that account for other key environmental factors, including environmental toxins and other forms of support, appear to be needed to further elucidate important associations with these trajectories. Such data could inform the development of more precise prevention approaches for a range of poor developmental outcomes and for the enhancement of brain development in children.

## Notes

Supplement. **eTable 1.** Variables Included in the ACEs Score **eTable 2.** Correlations Between Preschool and School-Age ACEs and Maternal Support Variables in Subjects with at Least One Scan **eTable 3.** Characteristics of Participants Included vs. Not Included in Analyses **eTable 4.** Separate General Linear Models of Scan 4 Brain Volumes by Preschool ACES and School-Age ACEs Covarying for Scan 4 Age and Sex **eTable 5.** General Linear Models of Scan 4 Hippocampus, Amygdala, Subgenual Cingulate, and Caudate Volume by Preschool ACEs and School-Age ACEs Covarying for Scan 4 Age and Sex (N=119) **eTable 6.** Separate General Linear Models of Scan 4 Brain Volumes by Preschool Maternal Support and School-Age Maternal Support Covarying for Scan 4 Age and Sex **eTable 7.** General Linear Models of Scan 4 Insula, Amygdala, and Caudate Volume by Preschool Maternal Support and School-Age Maternal Support Covarying for Scan 4 Age and Sex (N=101) **eFigure.** Study Flow
