# Supplementary material for: Association of Timing of Adverse Childhood Experiences and Caregiver Support With Regionally Specific Brain Development in Adolescents
Source: JAMA Netw Open. 2019 Sep 18;2(9):e1911426. doi: 10.1001/jamanetworkopen.2019.11426 (PMC6751767; doi:10.1001/jamanetworkopen.2019.11426)
Supplement: Supplement. — eTable 1. Variables Included in the ACEs Score eTable 2. Correlations Between Preschool and School-Age ACEs and Maternal Support Variables in Subjects with at Least One Scan eTable 3. Characteristics of Participants Included vs. Not Included in Analyses eTable 4. Separate General Linear Models of Scan 4 Brain Volumes by Preschool ACES and School-Age ACEs Covarying for Scan 4 Age and Sex eTable 5. General Linear Models of Scan 4 Hippocampus, Amygdala, Subgenual Cingulate, and Caudate Volume by Preschool ACEs and School-Age ACEs Covarying for Scan 4 Age and Sex (N=119) eTable 6. Separate General Linear Models of Scan 4 Brain Volumes by Preschool Maternal Support and School-Age Maternal Support Covarying for Scan 4 Age and Sex eTable 7. General Linear Models of Scan 4 Insula, Amygdala, and Caudate Volume by Preschool Maternal Support and School-Age Maternal Support Covarying for Scan 4 Age and Sex (N=101) eFigure. Study Flow [file jamanetwopen-2-e1911426-s001.pdf]

Supplementary Online Content

Luby JL, Tillman R, Barch DM. Association of timing of adverse childhood experiences and caregiver support with regionally specific brain development in adolescents. *JAMA Netw Open*. 2019;2(9):e1911426. doi:10.1001/jamanetworkopen.2019.11426

eTable 1. Variables Included in the ACEs Score
eTable 2. Correlations Between Preschool and School-Age ACEs and Maternal Support Variables in Subjects With at Least One Scan
eTable 3. Characteristics of Participants Included vs. Not Included in Analyses
eTable 4. Separate General Linear Models of Scan 4 Brain Volumes by Preschool ACES and School-Age ACEs Covarying for Scan 4 Age and Sex
eTable 5. General Linear Models of Scan 4 Hippocampus, Amygdala, Subgenual Cingulate, and Caudate Volume by Preschool ACEs and School-Age ACEs Covarying for Scan 4 Age and Sex (N=119)
eTable 6. Separate General Linear Models of Scan 4 Brain Volumes by Preschool Maternal Support and School-Age Maternal Support Covarying for Scan 4 Age and Sex
eTable 7. General Linear Models of Scan 4 Insula, Amygdala, and Caudate Volume by Preschool Maternal Support and School-Age Maternal Support Covarying for Scan 4 Age and Sex (N=101)
eFigure. Study Flow

This supplementary material has been provided by the authors to give readers additional information about their work.

© 2019 Luby JL et al. *JAMA Network Open*.

eTable 1. Variables Included in the ACEs Score

Poverty (Income-to-needs ratio < 1)
Traumatic life events
Parent arrest
Parent hospitalization
Crash with motor vehicle, plane, or boat
Accidental burning, poisoning, or drowning
Attacked by an animal
Death of adult loved one
Death of sibling or peer
Domestic violence*
Hospitalized, visited emergency department, or had invasive medical procedure
Man-made disaster
Natural disaster
Physical abuse
Sexual abuse, sexual assault, or rape
Witnessed someone threatened with harm, seriously injured, or killed<br>Physical violence or event causing death or severe harm**
Other traumatic life event
Parental psychiatric disorders
Parental suicidality
Parental substance use disorder
Other parental psychiatric disorder

*Assessed in the PAPA, not the CAPA
**Assessed in the CAPA, not the PAPA
All variables were coded as absent or present (0 vs 1) and adverse experiences (other than poverty) were only counted multiple times if they were non-redundant (e.g., child was abused at age 3 and again at age 4). ACEs variables were standardized (Z-scored) at each assessment wave, and then ACEs scores for preschool (ages 3.0-5.11) and school-age (6.0-8.11) were calculated by taking the mean of the ACEs variables from assessments occurring during those age ranges.

Coding Maternal Support Variables: More specifically, support was coded during an interval when parents expressed predominantly positive emotions during a task that was mildly stressful for the child with the parent present. When they remained calm, and when they were reassuring when reacting to the child's emotional expressions elicited by task support was coded as positive. Each instance of specific types of supportive caregiving strategies employed by the parent were counted as 1 unit and summed to give an overall preschool maternal support score. The support score represents the total number of supportive behaviors the caregiver was observed using over the course of the 8-minute task.

Puzzle Task Coding: The 5-minute task was separated into 30-second segments during which coders trained to reliability provided ratings for the frequency of parents' supportive caregiving behaviors observed during the task. Imaging Methods: For approximately 10% of sessions, poor scan quality (in both MPRAGEs) required excluding those sessions from the longitudinal analysis (n = 29, 21, 17, and 2 at the four waves, respectively). In those cases, FreeSurfer's longitudinal stream was run using the remaining available sessions for that participant.

FreeSurfer Variables:
Volumes of the anterior insula (G_insular_short_volume + S_circular_insula_ant_vol), subgenual cingulate (G_rectus_volume + G_subcallosal_volume), dorsolateral prefrontal cortex (G_front_middle_volume + S_front_middle_volume), dorsal anterior cingulate (G_and_S_cingul_Mid_Ant_vo), and rostral cingulate (G_and_S_cingul_Ant_volume) were taken from Destrieux Atlas [33].

© 2019 Luby JL et al. *JAMA Network Open*.

eTable 2. Correlations Between Preschool and School-Age ACEs and Maternal Support Variables in Subjects with at Least One Scan

	Preschool ACEs	School-age ACEs	Preschool support	School-age support
Preschool ACEs	1.000<br>N=164			
School-age ACEs	0.705<br>p<0.001<br>N=159	1.000<br>N=169		
Preschool support	-0.056<br>p=0.50<br>N=151	-0.100<br>p=0.23<br>N=147	1.000<br>N=152	
School-age support	-0.233<br>p=0.005<br>N=141	-0.216<br>p=0.01<br>N=139	0.391<br>p<0.001<br>N=134	1.000<br>N=142

© 2019 Luby JL et al. *JAMA Network Open*.

eTable 3. Characteristics of Participants Included vs. Not Included in Analyses

	Included (N=164)			Not Included* (N=47)			Included vs. Not Included	
**Characteristic**	**Total N**	**%**	**N**	**Total N**	**%**	**N**	**χ^2**	**p**
Male sex	164	51.2	84	47	48.9	23	0.08	0.78
Race	164			47			0.97	0.62
Caucasian		53.7	88		46.8	22		
African-American		34.8	57		42.6	20		
Other		11.6	19		10.6	5		
	**Total N**	**Mean**	**SD**	**Total N**	**Mean**	**SD**	**t**	**p**
Scan 1 age	164	10.28	1.24	47	10.29	1.35	-0.07	0.95
Scan 2 age	159	11.82	1.16	43	11.52	1.35	1.45	0.15
Scan 3 age	143	13.10	1.20	37	12.49	1.43	2.62	0.01
Scan 4 age	137	16.46	1.00	35	15.71	1.49	2.82	0.007
Scan 1 income-to-needs ratio	164	1.71	1.00	47	1.70	0.80	0.08	0.94
Preschool ACEs	164	0.13	1.00	4	-0.23	0.64	0.72	0.47
School-age ACEs	159	0.09	1.05	13	-0.10	0.71	0.65	0.52
Preschool maternal support	151	11.87	8.57	4	8.00	6.58	0.89	0.37
School-age maternal support	141	30.72	9.09	5	26.90	11.49	0.91	0.36

*Subjects were not included in analyses if they had unusable scan data at all scan waves or were missing preschool ACEs data, as this was an independent variable in every multilevel model

© 2019 Luby JL et al. *JAMA Network Open*.

eTable 4. Separate General Linear Models of Scan 4 Brain Volumes by Preschool ACES and School-Age ACEs Covarying for Scan 4 Age and Sex

**IV: Preschool ACEs (N=123)**	**Est.**	**SE**	**t**	**p**	**FDR p**	**ES**
DV: Insula Volume	-0.123	0.033	-3.78	<0.001	0.002	0.11
DV: Hippocampus Volume	-0.102	0.040	-2.53	0.01	0.03	0.05
DV: Amygdala Volume	-0.043	0.017	-2.58	0.01	0.03	0.05
DV: Subgenual Cingulate Volume	-0.106	0.040	-2.66	0.009	0.03	0.06
DV: Dorsal Prefrontal Volume	-0.076	0.165	-0.46	0.64	0.64	0.00
DV: Dorsal Cingulate Volume	-0.042	0.043	-0.98	0.33	0.37	0.01
DV: Rostral Cingulate Volume	-0.126	0.070	-1.81	0.07	0.10	0.03
DV: Caudate Volume	-0.101	0.043	-2.32	0.02	0.04	0.04
DV: Putamen Volume	-0.117	0.055	-2.13	0.04	0.06	0.04
DV: Nucleus Accumbens Volume	-0.012	0.010	-1.21	0.23	0.28	0.01
**IV: School-Age ACEs (N=124)**	**Est.**	**SE**	**t**	**p**	**FDR p**	**ES**
DV: Insula Volume	-0.106	0.032	-3.35	0.001	0.01	0.09
DV: Hippocampus Volume	-0.084	0.039	-2.14	0.03	0.17	0.04
DV: Amygdala Volume	-0.027	0.016	-1.68	0.10	0.19	0.02
DV: Subgenual Cingulate Volume	-0.059	0.039	-1.51	0.13	0.22	0.02
DV: Dorsal Prefrontal Volume	-0.005	0.159	-0.03	0.97	0.97	0.00
DV: Dorsal Cingulate Volume	-0.044	0.043	-1.03	0.30	0.38	0.01
DV: Rostral Cingulate Volume	-0.120	0.068	-1.76	0.08	0.19	0.03
DV: Caudate Volume	-0.040	0.043	-0.91	0.36	0.40	0.01
DV: Putamen Volume	-0.101	0.053	-1.90	0.06	0.19	0.03
DV: Nucleus Accumbens Volume	-0.012	0.010	-1.24	0.22	0.31	0.01

ES = effect size, partial eta-squared

© 2019 Luby JL et al. *JAMA Network Open*.

eTable 5. General Linear Models of Scan 4 Hippocampus, Amygdala, Subgenual Cingulate, and Caudate Volume by Preschool ACEs and School-Age ACEs Covarying for Scan 4 Age and Sex (N=119)

**DV: Hippocampus Volume**	**Est.**	**SE**	**t**	**p**
Intercept	4.808	0.061	78.92	<0.001
Female sex	-0.394	0.085	-4.66	<0.001
Scan 4 age	0.025	0.042	0.59	0.56
Preschool ACEs	-0.103	0.059	-1.74	0.08
School-age ACEs	-0.013	0.058	-0.23	0.82
**DV: Amygdala Volume**	**Est.**	**SE**	**t**	**p**
Intercept	1.751	0.025	70.90	<0.001
Female sex	-0.244	0.034	-7.13	<0.001
Scan 4 age	0.016	0.017	0.97	0.34
Preschool ACEs	-0.067	0.024	-2.77	0.007
School-age ACEs	0.022	0.023	0.93	0.35
**DV: Subgenual Cingulate Volume**	**Est.**	**SE**	**t**	**p**
Intercept	3.180	0.061	51.89	<0.001
Female sex	-0.235	0.085	-2.76	0.007
Scan 4 age	0.038	0.042	0.90	0.37
Preschool ACEs	-0.121	0.060	-2.03	0.04
School-age ACEs	0.026	0.058	0.44	0.66
**DV: Caudate Volume**	**Est.**	**SE**	**t**	**p**
Intercept	3.947	0.066	59.57	<0.001
Female sex	-0.071	0.092	-0.77	0.44
Scan 4 age	-0.028	0.046	-0.58	0.56
Preschool ACEs	-0.165	0.065	-2.55	0.01
School-age ACEs	0.082	0.063	1.31	0.19

© 2019 Luby JL et al. *JAMA Network Open*.

eTable 6. Separate General Linear Models of Scan 4 Brain Volumes by Preschool Maternal Support and School-Age Maternal Support Covarying for Scan 4 Age and Sex

**IV: Preschool Support (N=116)**	**Est.**	**SE**	**t**	**p**	**FDR p**	**ES**
DV: Insula Volume	0.004	0.004	0.99	0.32	0.54	0.01
DV: Hippocampus Volume	0.017	0.005	3.38	0.001	0.005	0.09
DV: Amygdala Volume	0.005	0.002	2.23	0.03	0.09	0.04
DV: Subgenual Cingulate Volume	0.002	0.005	0.47	0.64	0.64	0.00
DV: Dorsal Prefrontal Volume	0.014	0.020	0.70	0.48	0.59	0.00
DV: Dorsal Cingulate Volume	-0.003	0.005	-0.63	0.53	0.59	0.00
DV: Rostral Cingulate Volume	0.007	0.009	0.80	0.43	0.59	0.01
DV: Caudate Volume	0.019	0.005	3.57	<0.001	0.005	0.10
DV: Putamen Volume	0.012	0.007	1.67	0.10	0.19	0.02
DV: Nucleus Accumbens Volume	0.002	0.001	1.68	0.10	0.19	0.02
**IV: School-Age Support (N=107)**	**Est.**	**SE**	**t**	**p**	**FDR p**	**ES**
DV: Insula Volume	0.011	0.004	2.72	0.008	0.04	0.07
DV: Hippocampus Volume	0.015	0.005	3.03	0.003	0.03	0.08
DV: Amygdala Volume	0.005	0.002	2.58	0.01	0.04	0.06
DV: Subgenual Cingulate Volume	0.004	0.005	0.77	0.44	0.62	0.01
DV: Dorsal Prefrontal Volume	0.006	0.020	0.30	0.76	0.76	0.00
DV: Dorsal Cingulate Volume	0.003	0.005	0.56	0.58	0.64	0.00
DV: Rostral Cingulate Volume	0.010	0.008	1.22	0.23	0.38	0.01
DV: Caudate Volume	0.011	0.005	2.09	0.04	0.10	0.04
DV: Putamen Volume	0.011	0.007	1.63	0.11	0.21	0.03
DV: Nucleus Accumbens Volume	0.001	0.001	0.69	0.49	0.62	0.00

ES = effect size, partial eta-squared

© 2019 Luby JL et al. *JAMA Network Open*.

eTable 7. General Linear Models of Scan 4 Insula, Amygdala, and Caudate Volume by Preschool Maternal Support and School-Age Maternal Support Covarying for Scan 4 Age and Sex (N=101)

**DV: Insula Volume**	**Est.**	**SE**	**t**	**p**
Intercept	3.014	0.140	21.54	<0.001
Female sex	-0.335	0.079	-4.25	<0.001
Scan 4 age	-0.016	0.041	-0.39	0.70
Preschool maternal support	0.001	0.005	0.15	0.88
School-age maternal support	0.011	0.005	2.36	0.02
**DV: Amygdala Volume**	**Est.**	**SE**	**t**	**p**
Intercept	1.598	0.072	22.22	<0.001
Female sex	-0.254	0.041	-6.27	<0.001
Scan 4 age	-0.019	0.021	-0.90	0.37
Preschool maternal support	0.003	0.003	1.05	0.29
School-age maternal support	0.005	0.002	1.97	0.05
**DV: Caudate Volume**	**Est.**	**SE**	**t**	**p**
Intercept	3.553	0.174	20.44	<0.001
Female sex	-0.155	0.098	-1.58	0.12
Scan 4 age	-0.054	0.051	-1.07	0.29
Preschool maternal support	0.019	0.006	2.94	0.004
School-age maternal support	0.007	0.006	1.22	0.23

© 2019 Luby JL et al. *JAMA Network Open*.

eFigure. Study Flow: Scan 2 was approx. 18 months after Scan 1, Scan 3 was 15 months after S2, Scan 4 was 41 months after S3 (due to time between grant cycles)

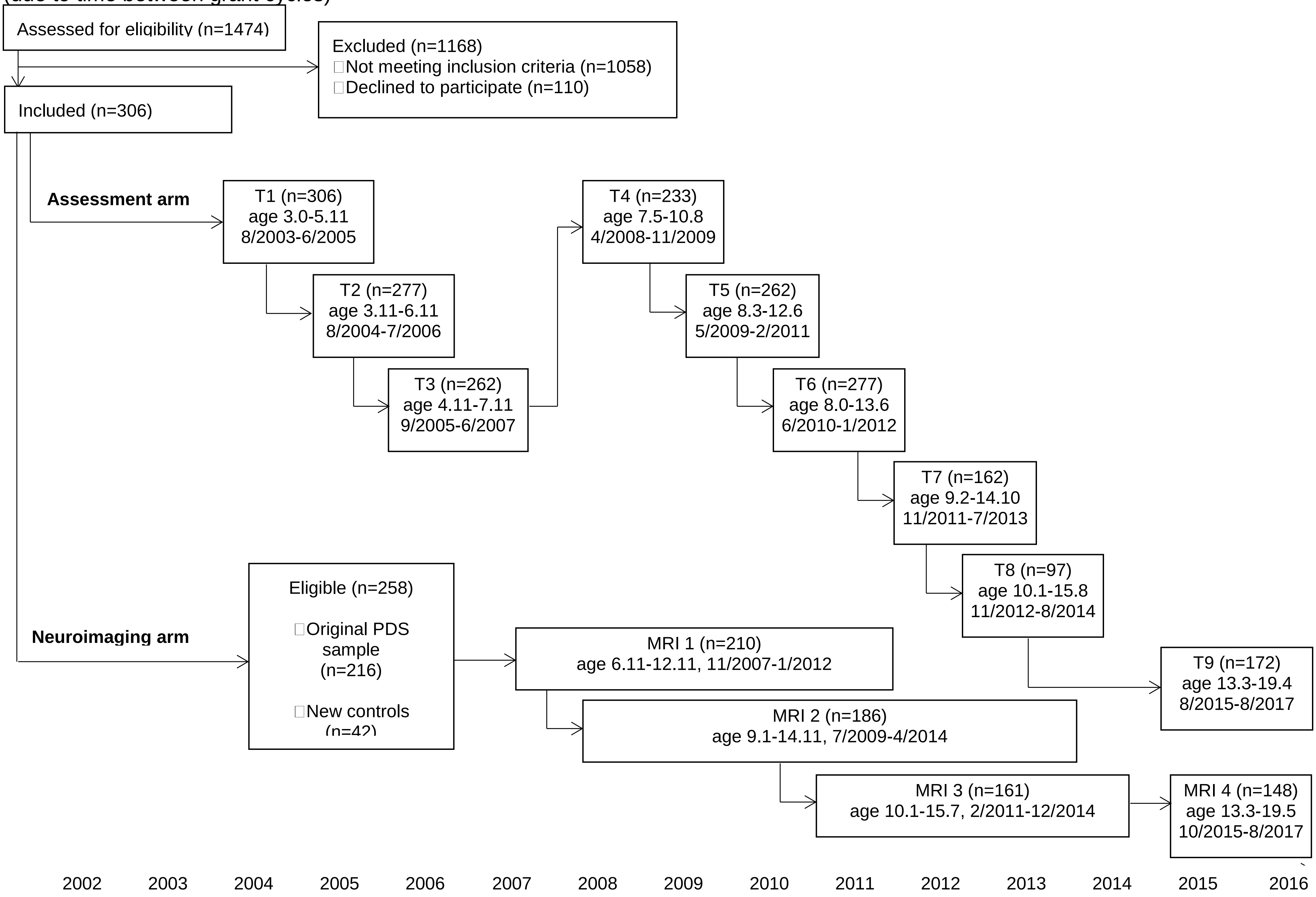

© 2019 Luby JL et al. *JAMA Network Open*.
